# Amelioration of ultraviolet-induced photokeratitis in mice treated with astaxanthin eye drops

**Published:** 2012-02-14

**Authors:** Anton Lennikov, Nobuyoshi Kitaichi, Risa Fukase, Miyuki Murata, Kousuke Noda, Ryo Ando, Takeshi Ohguchi, Tetsuya Kawakita, Shigeaki Ohno, Susumu Ishida

**Affiliations:** 1 Laboratory of Ocular Cell Biology and Visual Science, Department of Ophthalmology, Hokkaido University Graduate School of Medicine, Sapporo, Japan; 2 Department of Ocular Inflammation and Immunology, Hokkaido University Graduate School of Medicine, Sapporo, Japan; 3 Department of Ophthalmology, Health Sciences University of Hokkaido, Sapporo, Japan; 4 Department of Ophthalmology, Keio University, School of Medicine, Tokyo, Japan

## Abstract

**Purpose:**

Ultraviolet (UV) acts as low-dose ionizing radiation. Acute UVB exposure causes photokeratitis and induces apoptosis in corneal cells. Astaxanthin (AST) is a carotenoid, present in seafood, that has potential clinical applications due to its high antioxidant activity. In the present study, we examined whether topical administration of AST has preventive and therapeutic effects on UV-photokeratitis in mice.

**Methods:**

C57BL/6 mice were administered with AST diluted in polyethylene glycol (PEG) in instillation form (15 μl) to the right eye. Left eyes were given vehicle alone as controls. Immediately after the instillation, the mice, under anesthesia, were irradiated with UVB at a dose of 400 mJ/cm^2^. Eyeballs were collected 24 h after irradiation and stained with H&E and TUNEL. In an in vitro study, mouse corneal epithelial (TKE2) cells were cultured with AST before UV exposure to quantify the UV-derived cytotoxicity.

**Results:**

UVB exposure induced cell death and thinning of the corneal epithelium. However, the epithelium was morphologically well preserved after irradiation in AST-treated corneas. Irradiated corneal epithelium was significantly thicker in eyes treated with AST eye drops, compared to those treated with vehicles (p<0.01), in a doses dependent manner. Significantly fewer apoptotic cells were observed in AST-treated eyes than controls after irradiation (p<0.01). AST also reduced oxidative stress in irradiated corneas. The in vitro study showed less cytotoxicity of TKE2 cells in AST-treated cultures after UVB-irradiation (p<0.01). The cytoprotective effect increased with the dose of AST.

**Conclusions:**

Topical AST administration may be a candidate treatment to limit the damages by UV irradiation with wide clinical applications.

## Introduction

Ultraviolet (UV) irradiation represents a significant environmental hazard that can cause acute and chronic inflammatory changes in the cornea, lens, and retina of the eye. The sources of UV radiation are not merely from electric welding and tanning lamps but also from sunny days on the sea or in snowy mountains when eyes are left unprotected. In recent decades, the risk of acute photochemically-induced ocular damage has increased due to stratospheric ozone depletion [1]. UVB exposure causes photokeratitis, which is associated with expression of nuclear factor (NF)-κB, prostaglandin E2 (PGE2), and many other inflammatory agents [1]. Acute UVB exposure causes damage deeper than the epithelium, involving all tissues of the cornea [2] and inducing apoptosis in corneal cells [3,4]. Although the energy is much less than that of the gamma rays, ophthalmologists find it is necessary to study ways to prevent the damages caused by UV radiation due to its association with clinical ocular diseases. We have already reported that applying UVB irradiation at 400 mJ/cm^2^ to mice corneas can be a useful model for studying corneal inflammation [3].

Antioxidant enzymes, such as superoxide dismutase [5] and catalase [6], and pharmacologic antioxidants like N-acetyl cysteine [7,8], inhibit tumor necrosis factor (TNF)-α activation through NF-κB-dependent gene expression [9]. Astaxanthin (AST), 3,30-dihydroxy-b,b-carotene-4,40-dione, a carotenoid without vitamin A activity [10,11], has potential clinical applications due to its antioxidant activity, which is higher than β-carotene and α-tocopherol [10,12,13]. In addition, it has many highly potent pharmacological effects, including anti-tumor, anti-cancer, anti-diabetic, and anti-inflammation activities [12,14-16]. The potent activity of AST has been observed to modulate biologic functions ranging from lipid peroxidation to tissue protection [14,17]. The presence of the hydroxyl (OH) and keto (C=O) on each ionone ring in AST explains its unique feature of antioxidant activity for the protection of both the inner and outer membrane surfaces [10,14,18,19]. AST is found abundantly in the red-orange pigment of marine animals such as salmon (and salmon roe) and the shell of crabs and shrimp. AST and AST-like products are commonly indicated as antioxidants [20] and immune modulators [21]. One of the effects of AST is to scavenge reactive oxygen species [22]. We previously reported that AST showed a dose-dependent anti-inflammatory effect [23,24]. It was also reported that AST inhibited the production of inflammatory mediators by blocking NF-κB activation in vitro [4]. In the present study, we examined whether topical administration of AST has therapeutic effects on UV photokeratitis in mice.

## Methods

### Animals and reagents

Six- to 8-week-old C57BL/6 male mice were obtained from Clear Japan (Tokyo, Japan). Mice were maintained under specific pathogen-free conditions. All procedures involving animals were performed in accordance with the ARVO resolution on the use of animals in research. AST was purchased from Sigma-Aldrich (Tokyo, Japan).

### UV irradiation

The mice were administered with AST diluted in polyethylene glycol (PEG) in instillation form (15 μl) to the right eye once at the following concentrations: 1, 0.1, and 0.01 mg/ml (10 mice per group. Left eyes were instilled with vehicle alone. Immediately after the instillation, anesthetized mice were irradiated with UVB at a dose of 400 mJ/cm^2^ from a FS-20 (Panasonic, Osaka, Japan) fluorescent lamp. These bulbs have a broad emission spectrum (250–400 nm) with high output primarily in the UVB spectrum (290–320 nm). To evaluate the effects of AST on the corneal surface without UVB damage, control mice received the instillation of 1 mg/ml of AST without irradiation. All experiments were performed in triplicate.

### H&E and TUNEL staining

The eyes were dissected from mice 24 h after UVB exposure and fixed with 4% paraformalin. Tissue sections were prepared and stained with hematoxylin-eosin (H&E) for morphological analysis. Other sections were stained by terminal deoxynucleotidyl transferase dUTP nick end labeling (TUNEL) assay to detect apoptotic signaling. Apoptotic cells were detected with a Cell Death Detection Kit (Roche Diagnostics Japan, Tokyo) containing all necessary reagents for staining. Slides imaging, cell counting, and thickness evaluations were performed with BZ-9000 fluorescence microscope (Keyence, Japan) and software bundled with the apparatus. At least 10 sections were used to evaluate the epithelial thickness, measurements of 10 randomly selected areas of epithelium of central cornea was performed and averaged.

### Evaluation of the “sunglasses effect” of AST

The “sunglass effect” represents the status when protective properties of a substance are only due to blocking the UV rather than having a cytoprotective effect. To evaluate this effect, and whether AST is capable of rescuing the damaged epithelia after UVB irradiation, we performed additional experiments where mice were first irradiated with UVB at a dose of 400 mJ/cm^2^ under anesthesia. After 5 min of irradiation, the right eyes were then instilled with AST (1 mg/ml) and the left eyes instilled with vehicle alone.

### Detection of reactive oxygen species (ROS)

The eyes were dissected from mice 24 h after UVB exposure and frozen fresh in optimal cutting temperature (OCT) compound with liquid nitrogen. To visualize not only oxidative signaling but also the tissue structure, 4',6-diamidino-2-phenylindole (DAPI) dye (blue) was used to stain the nuclei of the cells. Dihydroethidium (DHE; Sigma-Aldrich, St. Louis, MO), an oxidative fluorescent dye, was used for the immunohistochemical detection of cytosolic superoxide anion (O_2_^-^) to evaluate ROS production in corneal epithelium tissue, as reported recently [25].

### NF-κB immunohistochemistry staining

The eyes were dissected from mice 24 h after UVB exposure and fixed with 4% paraformaldehyde and then paraffin-embedded. To evaluate the NF-κB positive cells in the corneal epithelium, slides were rehydrated and applied with a 1:50 dilution of NF-κB P65 antibody (Santa Cruz Biotechnology, Santa Cruz, CA) for 12 h, washed with PBS and then applied with 1:100 dilution of secondary goat anti–rabbit antibody dye conjugate (Invitrogen, Carlsbad, CA), which gives red fluorescence. Since NF-κB is widely present in intercellular tissue, to detect its expression in cell nuclei, we stained the section slides with 1:100 dilution of YO-PRO®-1 (Invitrogen), which produces a green signal from the cell nuclei. NF-κB positive (yellow) nuclei were quantified in merged images.

### In vitro study

TKE2 cells were used for in vitro study. TKE2 is a murine corneal epithelium-derived progenitor cell line [26,27]. Mouse TKE2 cells were cultured in Defined K-SFM (Keratinocye Serum Free Medium) with L-Glutamine, Phenol Red, and Sodium Pyruvate (Invitrogen), supplemented with 100 U/ml of penicillin and 100 U/ml of streptomycin, (Invitrogen). The cells were seeded onto 24-well plates (5×10^4^ cells/well) at 37 °C in a humidified incubator containing 5% CO_2_. The cells were treated with 1, 0.1, or 0.01 mg/ml AST. After 6 h of incubation, the wells were irradiated with UVB at a dose of 300 mJ/cm^2^ at room temperature for 1 min. To prevent UVB radiation absorption, the culture media were removed just before irradiation and replaced with a thin layer of phosphate-buffered saline (PBS). After UVB irradiation, cells were fed with fresh medium. The LDH (lactate dehydrogenase) Cytotoxicity Detection Kit (Roche Applied Science, Penzberg, Germany) was used to determine cytotoxicity 6 h after UVB exposure. Culture supernatants (100 μl) were collected and added to the kit’s work solution. After 30 min incubation, the absorbance was measured with an ELISA reader. All procedures were performed according the user manual of the kit. Analysis was performed in triplicate. To determine the background LDH activity of the medium and spontaneous LDH release, additional plates were prepared with empty medium and unaffected cells. The maximum LDH release was achieved by adding 100 μl of Triton-B to the reference wells. Cytotoxicity percentages were calculated, and Triton-B treated wells with maximum LDH release were assumed to be 100%. All experiments were performed in triplicate with 5 or more wells for each group in each experiment.

### Statistical analysis

All data were expressed as the mean±standard deviation (SD) from the respective test or control groups of data. Statistical significances were determined by the paired *t*-test and non-parametric Mann–Whitney U-test. P values less than 0.05 were considered significant.

## Results

### Morphological properties

**Figure 1:**
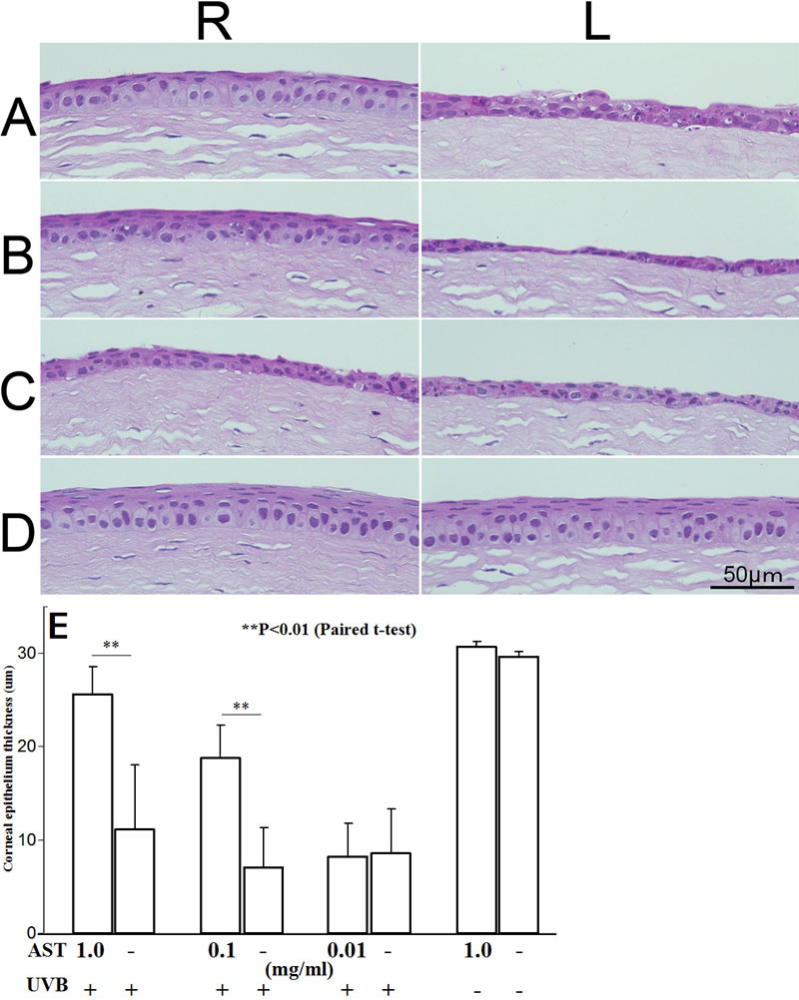
Morphological properties of ultraviolet (UV)-irradiated corneas. Eyes were treated with AST eye drops before UV exposure (**A**: 1 mg/ml, **B**: 0.1 mg/ml, **C**: 0.01 mg/ml AST). Control subjects were not irradiated with UVB (**D**). R: Right eyes were given various concentrations of AST eye drops. L: Left eyes were given vehicle alone as controls. The mean values of corneal epithelial thickness are summarized (E). Epithelia were significantly thicker in right eyes treated with 1 and 0.1 mg/ml AST compared to the left eyes, which served as controls (p<0.01). Protective effects of AST were found to occur in a dose-dependent manner.

We examined the morphological properties of UVB-irradiated and control corneas using H&E staining. At 24 h after UVB irradiation at a dose of 400 mJ/cm^2^, thinning and ulceration of the corneal epithelial layer were observed (Figure 1). The corneal epithelial thicknesses were 25.6±2.9, 18.8±3.5, and 8.2±3.6 μm in eyes treated with 1, 0.1, and 0.01 mg/ml of AST, respectively. The mean corneal epithelial thickness in eyes untreated with AST was 8.91±5.3 μm after UVB-exposure. The mean corneal epithelium thickness of non-irradiated eyes was 29.6±0.5 μm. Corneal epithelium was well preserved, and the thickness of the epithelium remained close to normal in the right eyes treated with 1 mg/ml of AST instillation (Figure 1A). Its protective effect decreased gradually in a concentration-dependent manner (Figure 1B,C). The corneas of mice administered with AST without UVB irradiation (Figure 1D) showed no differences from naïve corneas (Figure 1D). The mean values of the epithelium thickness of corneas were calculated and summarized (Figure 1E). Corneal epithelia were significantly thicker in eyes treated with 1 and 0.1 mg/ml AST eye drops compared with vehicle-treated eyes (p<0.01). Irradiated corneas treated with 1 mg/ml AST showed corneal epithelium thickness close to naïve cornea. AST protected corneal epithelium in a dose-dependent manner (Figure 1E).

**Figure 2:**
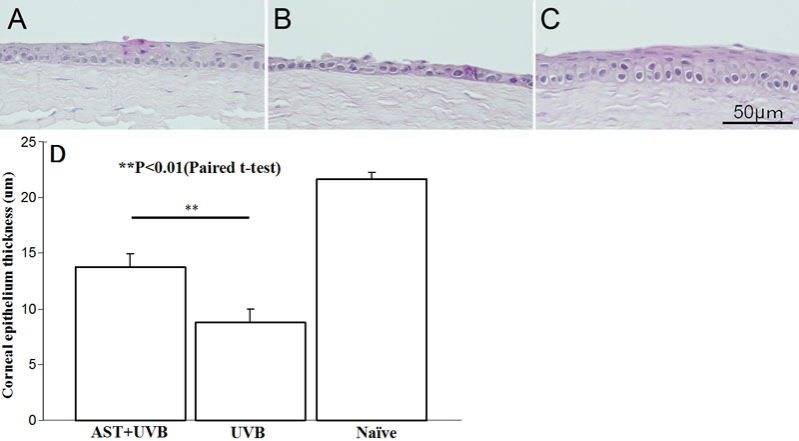
Morphological properties of ultraviolet (UV)-irradiated corneas treated with AST after irradiation. Eyes were treated with AST eye drops within 5 min after UVB exposure (**A**: 1 mg/ml, **B**: 0.1 mg/ml). Control subjects were not irradiated with UVB (**C**). The mean values of corneal epithelial thickness are summarized (**D**). Epithelia were significantly thicker when treated with 1 mg/ml AST compared to fellow eyes as controls (p<0.01). The protective effects of AST remained, even if eyes were treated after irradiation.

We also examined the morphological properties of UVB-irradiated and control corneas using H&E staining when AST was applied to corneas at a concentration of 1 mg/ml 5 min after UVB irradiation at a dose of 400 mJ/cm^2^. Twenty-four hours later, the corneal epithelial thickness was 13.7±0.8 μm (Figure 2A). The mean corneal epithelial thickness of vehicle-given corneas was 8.7±1.1 μm after UVB-exposure (Figure 2B). Non-irradiated corneal epithelium was 21.6±0.6 μm thick (Figure 2C). Even when AST was administered after UVB irradiation, the corneal epithelium was significantly thicker than in fellow eyes treated with vehicle (p<0.01, Figure 2D).

### TUNEL staining

**Figure 3:**
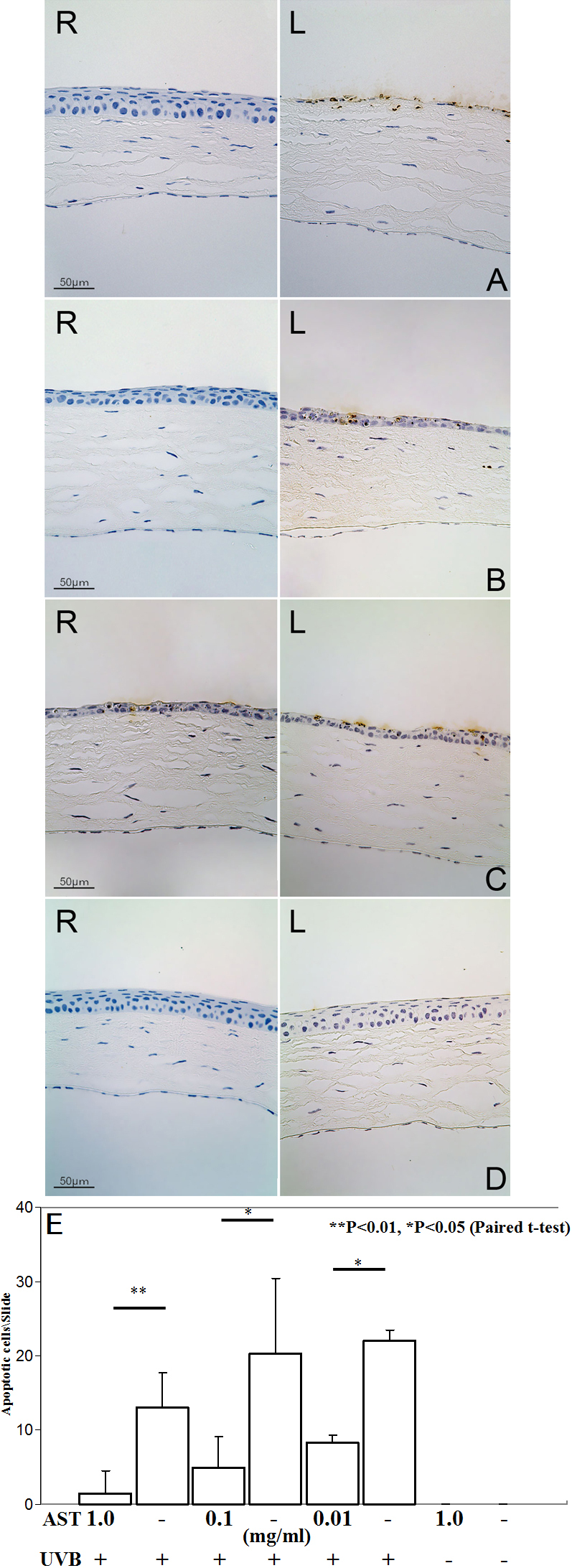
TUNEL labeling at irradiated corneas with and without AST-treatment. Eyes were treated with AST eye drops before UV exposure (**A**: 1 mg/ml, **B**: 0.1 mg/ml, **C**: 0.01 mg/ml AST). Control subjects were not irradiated with UVB (**D**). R: Right eyes were given various concentrations of AST eye drops. L: Left eyes were given vehicle alone as controls. Numbers of apoptotic corneal cells per slide after UVB exposure is shown (**E**). Apoptotic cells were significantly fewer in right eye corneas treated with 1 (p<0.01), 0.1 (p<0.05), and 0.01 (p<0.05) mg/ml AST eye drops compared to the left eyes, which served as controls. There were no apoptotic cells detected in corneas without irradiation.

We compared TUNEL staining in the corneas of AST-treated and untreated eyes of the mice 24 h after UV exposure. Few TUNEL-positive nuclei were detected in corneas of mice treated with AST at 1 and 0.1 mg/ml compared to untreated mice after UV irradiation (Figure 3A,B). No significant protective effect was observed in eyes treated with AST 0.01 mg/ml (Figure 3C). There were no apoptotic cells detected in corneas without UVB exposure (Figure 3D).

TUNEL-positive cells were counted, and the numbers were 1.5±3.0, 5.0±4.1, and 8.25±0.9 in eyes treated with 1, 0.1, and 0.01 mg/ml of AST, respectively (Figure 3E). The mean number of apoptotic cells was 18.4±4.7 in vehicle-given irradiated corneas. The apoptotic cells were significantly fewer in corneas treated with AST than those with vehicle only (p<0.01).

### Detection of reactive oxygen species (ROS)

**Figure 4:**
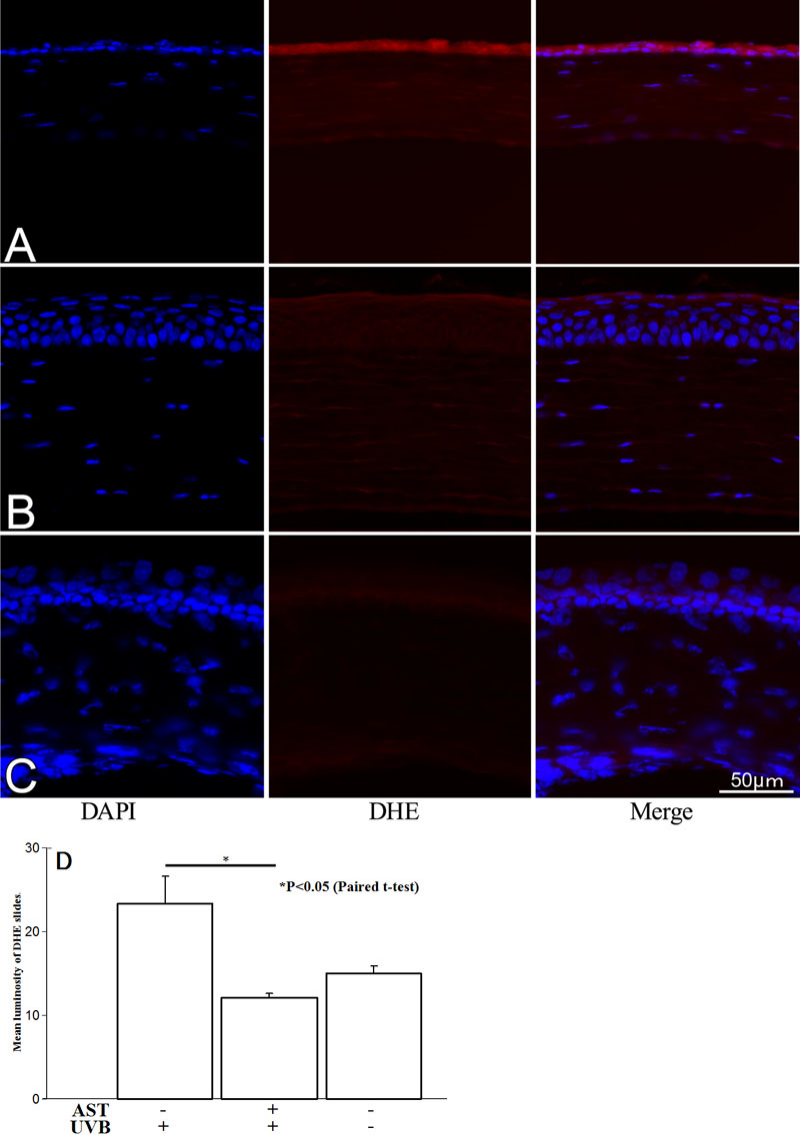
Reactive oxygen species signal expression after UVB exposure. Reactive oxygen species (ROS) were strongly detected (red) in untreated corneal epithelium after UVB irradiation (**A**). The ROS signal was weak in AST treated corneas (**B**) and close to unspecific ROS signaling in naïve corneas (**C**). The mean gray values of the corneal epithelium of DHE stained slides were evaluated by Image J software and summarized (**D**). Mean gray values were significantly lower in corneas treated with 1 mg/ml AST eye drops than in the vehicle-treated eyes (p<0.05).

Confocal microscopic images was examined to quantify reactive oxygen species (ROS) production in mouse corneal tissue. ROS production was determined by conversion of DHE to ethidium bromide (EtBr). All images were made in parallel at identical settings (Figure 4). ROS were strongly detected in untreated corneal epithelium after UVB irradiation (Figure 4A). However, the ROS signal was downregulated in AST-treated corneas (Figure 4B) and close to unspecific ROS signaling in naïve corneas (Figure 4C). The mean gray values of the corneal epithelium of DHE stained slides were evaluated by Image J software and summarized in Figure 4D. The mean gray value in vehicle-given irradiated corneas was 23.3±3.3. In eyes treated with 1 mg/ml of AST, after UVB irradiation, the mean gray value was 12.08±0.5. Non-irradiated corneal epithelium showed a 15.04±0.8 mean gray value. AST-treated epithelium showed significantly (p<0.05) lower mean gray values than irradiated corneas did.

### NF-κB downregulation by AST

**Figure 5:**
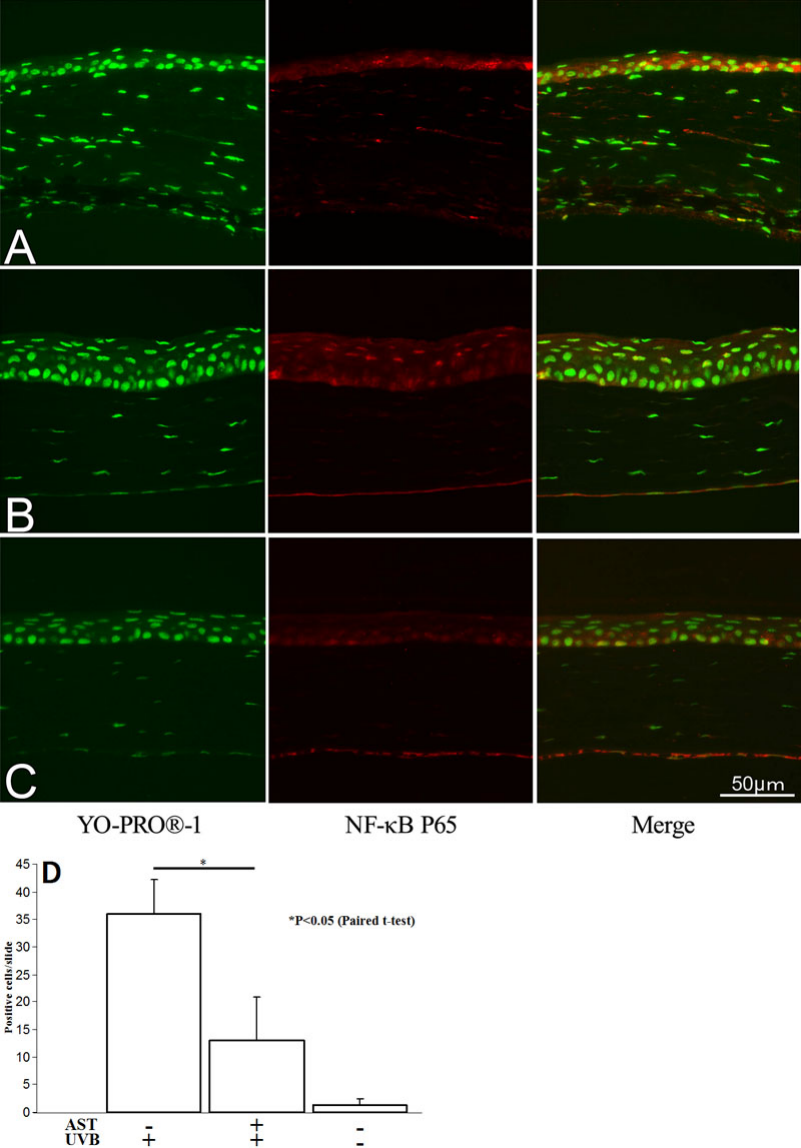
NF-κB expression in corneal epithelia. Eyes were given vehicle alone (**A**) or treated with AST eye drops before UV exposure (**B**); control subjects were not irradiated with UVB (**C**). The mean values of NF-κB positive cells (yellow) in corneal epithelial were summarized (**D**). Expression of NF-κB was significantly downregulated in AST-treated mice after UVB irradiation (p<0.05).

Previously, we reported that AST decreased NF-κB expression in endotoxin-induced uveitis (EIU) and choroidal neovascularization [23,24]. To examine whether AST administration effects NF-κB in corneal epithelium, we immunohistochemically analyzed AST expression in the collected corneal tissues and examined it with confocal microscope. NF-κB positive nuclei (yellow) were found to be 13.0±7.9 in corneal epithelial cells in UV-irradiated mice treated with AST at 1.0 mg/kg (Figure 5A). However, 36.0±6.2 (Figure 5B) of multiple NF-κB positive cells were found in the corneal epithelial cells in UV-irradiated mice untreated with AST. Naïve mice corneal tissues showed weak response to anti-NF-κB antibodies, and only 1.3±1.1 positive nuclei were found (Figure 5C). Expression of NF-κB was significantly downregulated in AST-treated mice after UVB irradiation (p<0.05, Figure 5D).

### AST suppressed phototoxicity in corneal epithelial cell cultures after UVB irradiation

**Figure 6:**
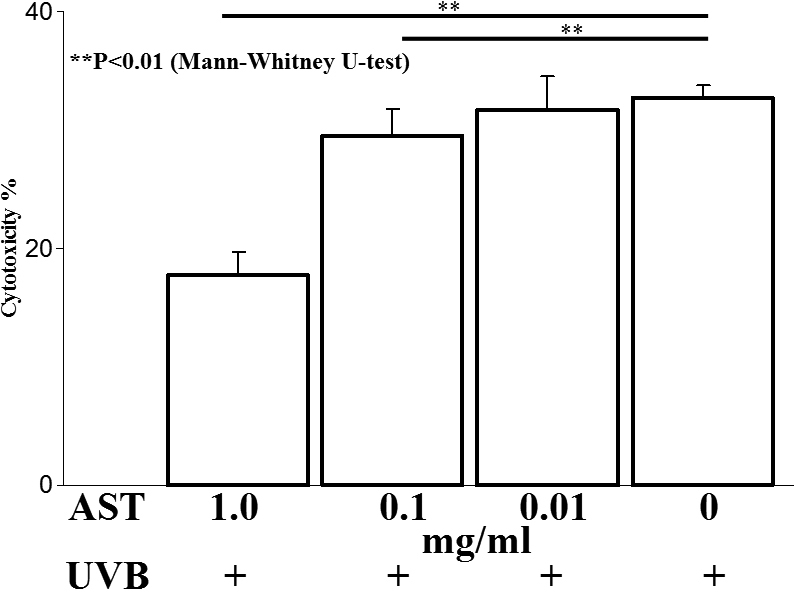
UVB-induced cytotoxicity in TKE2 cells in vitro. AST was added to a culture of UVB-irradiated TKE2 cells. Cytotoxicity after UVB exposure in keratinocyte cultures was significantly decreased by AST in a dose-dependent manner (1 mg/ml: p<0.01, 0.1 mg/ml: p<0.05, and 0.01 mg/ml: not significant).

Next, cytotoxicity was examined in UVB-irradiated TKE2 cells, murine corneal epithelium-derived progenitor cell line, treated or untreated with AST in vitro (Figure 6). The percentages of cytotoxicity after irradiation were 17.6±2.0, 29.5±2.2, 31.7.2±2.8, and 32.7±1.7% in wells containing 1, 0.1, 0.01, and 0 mg/ml of AST, respectively. Cytotoxicity was significantly suppressed by 1 mg/ml (p<0.01) and 0.1 mg/ml (p<0.05) AST administration. The effect was AST concentration-dependent.

## Discussion

The corneal epithelium serves to protect corneal structures against UV damage by absorbing a substantial amount of UV energy. Though the energy of UV is much less than that of ionizing radiation rays, the injuries on cells and DNA are critical. Excessive UVB irradiation induces various changes of DNA, proteins and cells, through activation of pro-inflammatory mediators including NF-κB. AST can form stable resonance structures by attachment of the carbonyl and hydroxyl groups to its β-ionone ring. It can also remove the chain-carrying lipid peroxyl radicals in the liposomal suspension, protecting cells from oxidative and free radical damages. NF-κB activates proinflammatory cytokines, chemokines, and enzymes that generate mediators of inflammation. It can also activate adhesion molecules that play a key role in the initial recruitment of leukocytes to sites of inflammation. Therefore, activation of NF-κB leads to a coordinated upregulation of many genes whose products mediate the inflammatory loop and perpetuate local inflammatory responses. In our previous studies, AST inhibited the in vivo activation of NF-κB in endotoxin-induced uveitis (EIU) and choroidal neovascularization [23,24]. Therefore, reactive oxygen species-induced oxidative stress may play an important role in NF-κB activation and proinflammatory cytokine production. Our immunohistochemical finding shows that AST has the same effects of reducing NF-κB expression in corneal epithelia. The anti-inflammatory effects of AST, through its suppression of NF-κB activation, may be based on its antioxidant activity, as previous reports showed that several antioxidants efficiently inhibit NF-κB activation induced by lipopolysaccharides (LPS) in cell systems [28-33]. Therefore, these effects appear to be mediated by the powerful radical scavenger properties of the antioxidants, which apparently counteract reactive oxygen intermediates generated by NF-κB activation [30,34,35].

We also showed that the relieving effect of AST on UVB photokeratitis is not derived from light-interception (sunglasses effect) but its direct pharmacological effect (Figure 2). In fact, a previous study showed that AST eye drops did not delay the progression of UVB-induced cataract in rats when AST was not on lens [36]. In addition, UVB irradiation improved AST accumulation in green microalgae [8]. These results strongly suggest that AST can produce a protective effect against UV, and organisms have been using AST to save themselves from harmful UV light.

AST is found abundantly in the red-orange pigment of marine animals such as salmon (and salmon roe) and the shells of crabs and shrimp. Humans have taken in these food products since ancient times, and AST is now available as an oral supplement. Therefore, AST may be free of any harmful side effects. In the present study, we demonstrated that topical AST, instead of systemic administration, is effective in protecting the ocular surface against UV exposure with no adverse effects. Thus, in conclusion, AST might be a promising naturally-derived substance for protecting ocular surfaces from the damages caused by ultraviolet radiation.
